# Usability Evaluation of a Mixed Reality Platform in Pediatric Interventional Cardiology by Specialist Physicians: Mixed Methods Study

**DOI:** 10.2196/79278

**Published:** 2025-11-19

**Authors:** Catherine Chilute Chilanga, Andreas Jahnen, Linda Hafskjold

**Affiliations:** 1 Center for Health and Technology, University of South-Eastern Norway, Grønland 58, Drammen, 3045, Norway, +47 31 00 90 83; 2 Department of Optometry, Radiography and Lighting Design, Faculty of Health and Social Sciences, University of South-Eastern Norway, Kongsberg, Norway; 3 Luxembourg Institute of Science and Technology, Esch Sur Alzette, Luxembourg

**Keywords:** cardiology, congenital heart disease, mixed reality, pediatrics, interventional radiology

## Abstract

**Background:**

Immersive technologies such as virtual reality, augmented reality, and mixed reality are used in pediatric interventional cardiology (IC) to improve anatomical visualization and spatial understanding and support procedural precision. These technologies offer 3D representations of complex cardiac structures, which can aid in preprocedural planning, intraoperative navigation, and team communication. As these technologies gain traction in IC, understanding how medical specialists perceive their value is vital.

**Objective:**

This study assesses what IC specialist physicians consider valuable in immersive technologies by evaluating their experiences with *CardioVision*, a software designed for pediatric IC procedures.

**Methods:**

A mixed methods design was used, combining questionnaires and semi-structured interviews. Purposeful and snowball sampling was used to recruit specialist physicians with experience in IC. The recruited specialist physicians were asked to submit IC cases for software testing. These were uploaded to *CardioVision* by the developer, who also conducted the testing in person with the physicians at their designated clinical stations. A total of eight specialist physicians completed a pretest questionnaire, used the software, and thereafter completed a posttest questionnaire, which included System Usability Scale and Likert-scale questions on experience with the software. The interviews explored the specialist physicians’ experience with the software’s performance. Of the 8 participants, 6 further participated in the interviews. The interviews were conducted by two of the authors. The qualitative data were analyzed using inductive content analysis, involving subjective interpretation of textual material from the interviews. Following the approach outlined by Elo and Kyngäs, the process comprised three phases: preparation, organization, and reporting. In the preparation and organization phases, one author organized and categorized the interview data in an Excel sheet. The two authors then reviewed the material, compared emerging codes and themes, and reached consensus on the final main theme and subthemes for reporting.

**Results:**

In total, 8 questionnaires and 6 interviews were obtained from specialist physicians from Belgium, France, Germany, and Italy. Quantitative data from mean System Usability Scale scores showed that users felt confident using the system (mean 4.25). When using the software, the highest positive feedback was for visualization of structures, where all users (8/8, 100%) agreed. Overall, most participants (6/8, 75%) rated the system positively for decision-making support and 5/8 (63%) for complex case preparation. One participant (1/8, 12%) disagreed with its effectiveness in preparing complex cases, indicating mixed opinions in this area. In the interviews, emerging themes aligned with quantitative data to show that participants valued the software’s ease of use, high-quality visualization, and decision-making support. Additional benefits included use in medical education, interprofessional collaboration, and patient communication. Developer–clinician collaboration was emphasized as vital for effective integration into routine practice.

**Conclusions:**

Immersive technologies offer clinical value when aligned with user needs and integrated through collaborative development.

## Introduction

Interventional cardiology (IC) uses image-guided techniques to perform minimally invasive procedures for diagnosing and treating various cardiac diseases [1]. In pediatric IC, less invasive solutions for congenital heart disease (CHD) are reducing the need for open-heart surgery, enhancing patient care [2], and transforming the clinical practice of interventional cardiologists [3]. IC has progressed over the years, integrating advanced technologies to enhance visualization of anatomical structures in CHD [4]. Technologies such as virtual reality (VR), augmented reality (AR), and mixed reality (MR) are now being integrated to enhance visualization and procedural precision in cardiology [5,6]. VR creates a simulated 3D environment mimicking real-world scenarios, AR overlays medical imaging onto the physical world for real-time interaction [4], and MR combines digital elements with reality for dynamic, interactive engagement [7]. Integrating these immersive technologies with artificial intelligence (AI) and wearable headsets capable of displaying holographic 3D images enhances user interaction with digital content in real-world environments, further expanding their utility in medical imaging [8].

Immersive technologies have made it possible to treat a wider range of CHDs than was previously achievable [3]. Ionizing radiation is used during IC procedures, resulting in substantial patient radiation exposure, and when performed on pediatric or adolescent patients, minimizing radiation risks is crucial [9]. In the HARMONIC (health effects of cardiac fluoroscopy and modern radiotherapy in pediatrics) project [10], efforts have focused on optimizing interventional procedures in pediatric cardiology patients to enhance safety and minimize radiation exposure. In line with the goals of the HARMONIC project, an innovative immersive technology, *CardioVision,* to support IC for pediatric patients, was developed. *CardioVision* is a VR and MR software integrated with AI technology. It provides a 3D reconstructed heart model, giving cardiologists unprecedented visual insights into cardiac anatomy and function [11]. *CardioVision* was developed by a multidisciplinary team of computer scientists, medical physicists, and pediatric cardiologists, focusing on the preparation of complex, high-risk cases. Using web technologies, the platform can be accessed from standard computers to MR devices without installing dedicated software. The full components of the software’s system are outlined in the methods.

Immersive technologies can be classified into two types: those used by patients and those used by health care providers [12], with *CardioVision* designed for the latter. Several applications and benefits of VR and AR technologies in medical imaging have been reported, including enhanced image quality, assistance with procedure preplanning, and support for other uses, such as multidisciplinary discussions and patient education [4]. Iqbal et al [13], however, point out that a crucial barrier to implementing most immersive technologies in health care is the lack of seamless integration with existing systems, compounded by technical challenges and limited clinical evidence for its use in critical procedures. Lara Hernandez et al [14] in a systematic review study further state that digital health technologies in cardiology remain at early stages, classified as either *proof of concept* or *robust candidates* that are yet to be integrated into routine clinical workflow. This implies that clinicians currently cannot fully rely on these tools for routine decision-making or patient care until they are thoroughly tested and proven effective in real-world clinical settings. This underscores a significant research gap: the need to identify and validate the most effective clinical applications of immersive technologies to support their practical, trustworthy use in cardiology. Kouijzer et al [15] suggest that for the implementation of immersive technology in health care, it is essential to address the entire process, identifying barriers and applying coordinated, multilevel strategies to ensure effective implementation. In this regard, understanding the best clinical applications for these immersive technologies is vital.

In this study, we evaluate *CardioVision,* a software designed for busy clinical settings. *CardioVision* is designed with a flexible workflow in mind. It uses standard internet technologies available on most computers, making case creation and management straightforward. Web-based extended reality enables AI-powered visualizations to be displayed across a wide range of hardware, ensuring clinicians have easy access and optimal viewing quality.

Our current aim is to assess how pediatric interventional cardiologists and other specialist physicians with experience in IC procedures perceive immersive technologies as valuable by evaluating their use of *CardioVision*.

## Methods

### Study Design

**Figure 1.**
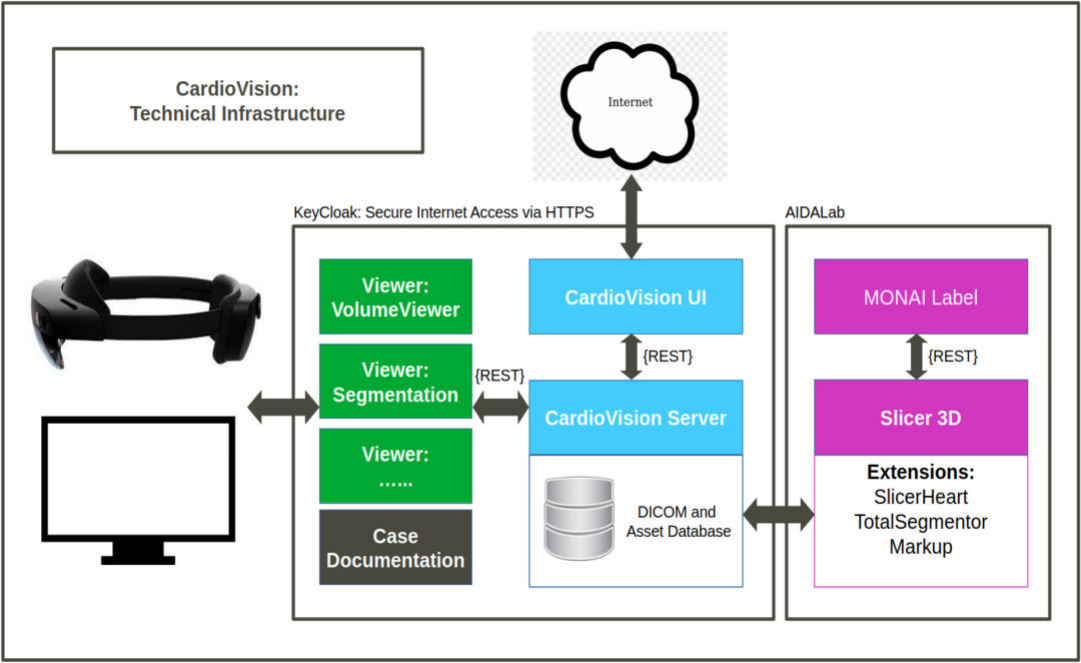
The *CardioVision* infrastructure provides data management (CardioVision UI and CardioVision Server), AI-enabled image processing (Slider 3D [17] and MONAI Label [18]), and viewing (volume, mixed reality, and viewers) components. All components are web-enabled to allow for central management, versatile deployment, and scalability.

The study used a mixed methods approach, combining data collection via questionnaires and semi-structured interviews. Specialist physicians with experience in IC were invited to test the *CardioVision*. The software system architecture is outlined in Figure 1. The system architecture is designed to enable seamless management and processing of medical imaging data using modern, responsive web technologies. A key advantage of this approach is that no dedicated client software needs to be installed on hospital workstations or 3D headsets. Users can upload anonymized patient data in Digital Imaging and Communications in Medicine format. While the platform primarily supports computed tomography (CT) scans, other modalities such as 3D ultrasound are also compatible. Each upload creates a new case that is securely stored on the *CardioVision* server.

The uploaded data is processed through a set of external tools for visualization and analysis. Clinicians can interact with the data through intuitive visualization tools, such as volume rendering, segmentation viewers, and a Kitware GLANCE-based viewer [16]. A demo account is available for testing on request to the developer (second author).

### Participants Recruitment

The sampling strategy aimed to recruit physicians with expertise in the field of IC and a strong interest in the adoption of XR technologies. Recruitment combined purposive sampling, which selects participants based on expertise and relevance [19], with snowball sampling, in which participants recommend others meeting the same criteria [20]. Initially, the purposive recruitment targeted physicians with experience in IC and who had an interest in evaluating the software. This step was conducted by the developer and author (AJ) using established professional networks to identify potential participants. The physicians were selected for their relevant expertise and motivation to participate and were invited to recommend colleagues with similar qualifications, completing recruitment through snowball sampling.

Eleven specialist physicians were invited to participate, of whom eight confirmed. Before the day of testing the software, each specialist physician selected and sent in a personal case that fell into the category of preparation for a difficult case. Table 1 indicates the type of cases submitted. The majority of cases were related to CHD, with the exception of a complex noncardiac aneurysm. No patient personal information was transmitted; only data sufficient to simulate the case for testing purposes were used to assess the software’s ease of use, the visualization of pathology, and the potential for radiation dose optimization.

**Table 1.** Cases assessed by the specialist physicians.

Case diagnosis	Description
Difficult Patent Ductus Arteriosus (PDA)	Large PDA in an infant unresponsive to medical therapy; required device-based closure.
Discontinuous pulmonary arteries	Rare congenital defect with separate origins of branch pulmonary arteries; required staged surgical reconstruction for unifocalization.
Complex malformation	The patient presented with a complex malformation of the heart, including several previous interventions.
Univentricular in a patient with double outlet	Double outlet right ventricle with univentricular physiology.
Aortic coarctation reduction	An aortic reduction occurs close to the right ventricle in an S shape.
Pulmonary artery stenosis	Branch pulmonary artery narrowing causing right ventricular pressure overload; treated with balloon angioplasty and stenting.
Angioplasty	Catheter-based dilation of stenotic vessel to restore blood flow.
Complex aneurysm	Noncardiac case showed a difficult-to-treat aneurysm. Visualizations include only the blood vessels.

**Figure 2.**
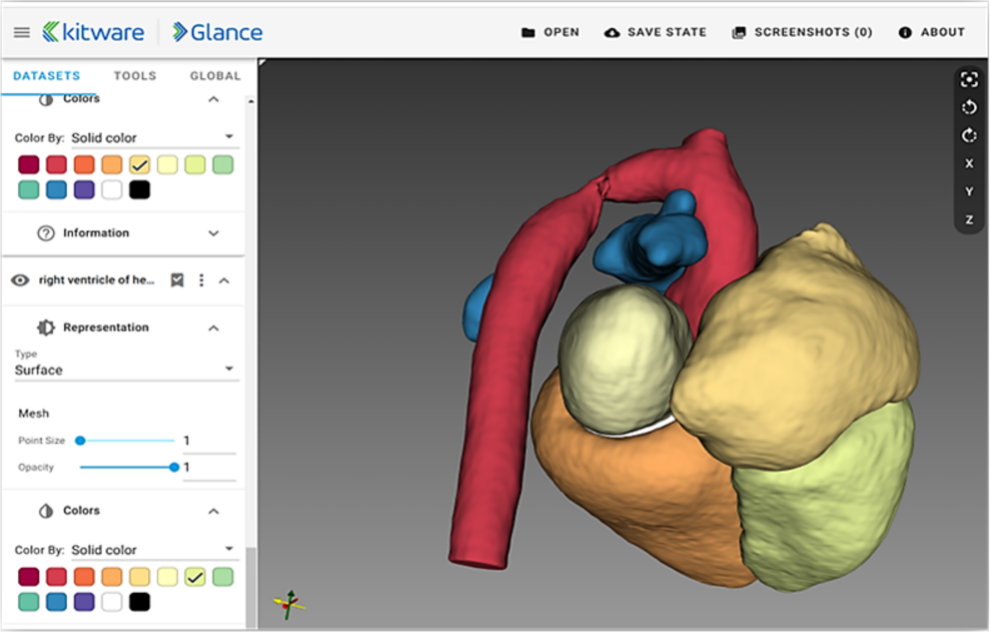
A sample visualization. The heart is segmented into different parts and colored for better differentiation.

The corresponding data to the cases were then processed and made available on the *CardioVision* software platform. Figures2  3 show examples of visualizations that help the cardiologist to decide on the best treatment.

**Figure 3.**
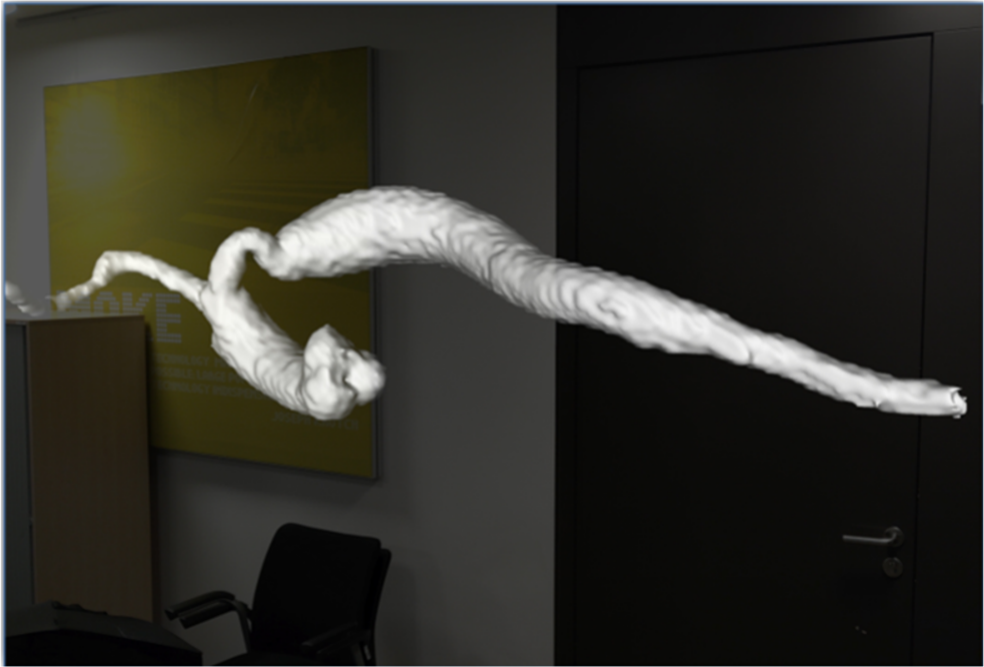
A segment of an aorta is shown in mixed reality. Due to the AI-based segmentation, the aorta is exempted. This allows us to see the reduction of the aorta and the S shape, which makes the treatment difficult.

Prior to using the *CardioVision* software, the specialist physicians completed a pre-questionnaire aimed at evaluating their expectations about its performance. After completion of the questionnaire on expectations, the specialist physicians were invited to use the software on the IC procedures. The software testing was performed with the developer physically present at the specialists’ clinical workstation or hospital. At the beginning of the testing, each specialist physician received a brief introduction to the *CardioVision* software platform and guidance on using the MR device (Microsoft HoloLens 2, Microsoft). After testing the software, the physicians answered a post-questionnaire, which included a standard 10-question System Usability Scale (SUS). The SUS is widely used as a standardized tool for evaluating perceived usability of systems and technologies [21]. The 10 questions (Qs) of the SUS are designed to assess various aspects of system usability. The items alternate between positive and negative statements to minimize response bias [21]. Positive statements (Q1, Q3, Q5, Q7, and Q9) focus on overall usability perception, system consistency, confidence in use, learnability, and integration into workflow. In contrast, negative statements (Q2, Q4, Q6, Q8, and Q10) address concerns related to complexity, need for technical support, inconsistency, cognitive load, and learnability challenges. Together, these questions provide a balanced evaluation of a system’s ease of use, user confidence, and potential barriers to adoption [21]. The 10 questions are outlined in the usability questionnaire (see attached questionnaire in Multimedia Appendix 1)

Additional questions about participants’ experiences were included following the SUS items to complement the usability assessment. Both the expectation and usability questionnaire had a 5-point Likert scale designed to assess the participants' agreements [22]. At the end of the questionnaire, the specialist physicians were asked to indicate if they agreed to further participate in an interview to describe their experience with the use of the software. Six of the 8 specialist physicians agreed to participate in the interviews.

Individual interviews were thereafter arranged for a later date at the participants' convenience and conducted using an interview guide (as shown in Multimedia Appendix 2). The first and last authors (CCC and LH) conducted the interviewers. The interview addresses five parts: initial impression of the software, experience of using it, the performance of the software, possible feature enhancements, and overall satisfaction of use. The interviews were recorded using the Nettskjema Diktafon app (University of Oslo, 2024), a secure tool for collecting audio data [23]. The interviews were conducted using a semi-structured guide and lasted approximately 25 to 40 minutes. The interview guide helped ensure that key topics related to the study’s aim were explored. Probing questions were used to explore the specialist physicians’ clinical examples and experiences, ensuring richness and depth of the data. Similar responses were observed after the first 3 interviews, and by the sixth participant, no new information had emerged. Saturation, defined as the point at which no new issues or insights emerge from subsequent interviews [24], is therefore assumed to have been reached. Achieving saturation is further supported by the sampling strategy, given that the selected participants possessed in-depth and broad insights into the key topics relevant to the study’s aim.

### Data Analysis

The quantitative data were analyzed using IBM SPSS Statistics version 29 (IBM Corp, 2022). The data were sorted in a Microsoft Excel file, coded, and exported to SPSS. Mean values were calculated to determine variation usability among the specialist physicians. In the qualitative data, inductive content analysis was used, which included preparing the data and organizing code to create themes [25]. This process comprised subjective interpretation of textual data and the classification of emerging codes to identify subthemes and main themes. The analysis followed the three phases of inductive content analysis as outlined by Elo and Kyngäs [25] of preparation, organization, and reporting. In the preparation phase, one of the researchers familiarized themselves with the data by reading through the interview transcripts and identifying relevant sections, which ranged from whole responses to selected words or phrases that captured key concepts. In the organization phase, individual codes were developed from the extracted text and subsequently grouped into subthemes and overarching main themes. The preparation and organizing of the qualitative data were sorted manually in a Microsoft Excel file. Two authors then jointly reviewed, discussed, and agreed upon the meanings and structure of the subthemes and main themes to ensure consistency and credibility. Finally, in the reporting phase, the results of the analysis were presented or reported. The quantitative and qualitative data were then triangulated to compare the different data sources. Explanatory sequential mixed methods were used where the quantitative data collection and analysis was followed by the collection of qualitative data, which are used to further explain the initial quantitative results [26].

### Ethical Considerations

Ethical approval for this study was obtained from the Norwegian Agency for Shared Services in Education and Research (reference number 441934). Written information about the study’s purpose, procedures, and confidentiality measures was provided to all specialist physicians prior to participation, and written informed consent was obtained electronically. As the evaluation of CardioVision involved the use of fully anonymized patient data for simulation and testing purposes only, the requirement for additional approval to use patient data was waived in accordance with the guidance of the Norwegian Agency for Shared Services in Education and Research. No identifiable patient information was collected, and the anonymized case data were not included in the study’s data analysis. Data collected from the participating specialist physicians were treated as strictly confidential and stored securely in compliance with applicable data protection regulations.

## Results

### Demographic Data of Participants

Eight specialist physicians from Belgium, France, Germany, and Italy, with experience in pediatric IC and interventional radiology, completed questionnaires, and 6 participated in individual interviews, resulting in 14 data sets. Table 2 shows an outline of the sample characteristics. A total of 5/8 (63%) specialist physicians had above 10 years’ experience in clinical practice. Half (4/8, 50%) of all the specialist physicians had experience with cardiac procedures as well as MR/VR use in clinical practice. Only 2 (2/8, 25%) specialist physicians, however, reported using MR/VR in cardiology. All the participants stated that they work in public practice. The 6 interviewed specialist physicians were all male: 4 working as pediatric interventional cardiologists and 2 as neurointervention surgeons. The participants interviewed were mostly those with over 10 years of professional experience in their respective fields, while only 1 had less than or equal to 5 years.

**Table 2.** Demographics and sample characteristics.

Participant (PT)	Interview	Specialty	Years of experience (y)	Pediatric cardiology experience	Familiar with MR^a^/VR^b^	Uses MR/VR in practice
PT1	Yes	Pediatric interventional cardiologist	>10	Yes	No	No
PT2	Yes	Pediatric interventional cardiologist	≤5	Yes	Yes	Yes
PT3	Yes	Pediatric interventional cardiologist	>10	Yes	No	No
PT4	Yes	Pediatric interventional cardiologist	>10	Yes	Yes	Yes
PT5	Yes	Neurointervention surgeon	>10	No	No	No
PT6	Yes	Neurointervention surgeon	>10	No	Yes	No
PT7	No	Neurointervention surgeon	6‐10	No	Yes	No
PT8	No	Neurointervention surgeon	1‐5	No	No	No

a MR, mixed reality.

b VR, virtual reality.

### System Usability

The SUS scores (Table 3) show that users feel confident using the system (Q5: 4.25 [range 3.00‐5.00]) and do not consider it overly complex (Q2: 1.88 [range 1.00‐3.00]). However, some users reported needing technical support (Q4: 3.00 [range 2.00‐5.00]), experiencing minor difficulties when learning to use the system (Q10: 2.37 [range 1.00‐4.00]), and finding it somewhat effortful to use (Q8:3.00 [range 1.00‐5.00]). The data from the SUS scores aligned with the interview data from the theme user experience, which is explained further below with the other emerging themes.

**Table 3.** SUS^a^ mean scores and statement interpretation of results.

SUS question	Mean (range^b^)	Interpretation
Q1 (positive)	3.75 (2.00‐5.00)	Users generally find the system usable, but there is room for improvement
Q2 (negative)	1.88 (1.00‐3.00)	The system is not perceived as overly complex
Q3 (positive)	3.75 (2.00‐5.00)	Users feel the system is fairly consistent in its design and behavior
Q4 (negative)	3.00 (2.00‐5.00)	Some users feel they might need technical support to use the system effectively
Q5 (positive)	4.25 (3.00‐5.00)	Users feel confident when using the system
Q6 (negative)	2.00 (1.00‐3.00)	The system is not perceived as highly inconsistent, but there might be some inconsistencies.
Q7 (positive)	3.75 (2.00‐5.00	Users generally find the system easy to learn
Q8 (negative)	3.00 (1.00‐5.00)	Some users find the system somewhat effortful to use, indicating potential usability barriers
Q9 (positive)	3.38 (2.00‐5.00)	The system fits reasonably well into users’ workflows but could be improved
Q10 (negative)	2.37 (1.00‐4.00)	Some users experience minor difficulties in learning how to use the system

a SUS, System Usability Scale.

b Minimum-maximum values.

### Themes Emerging From Interview Data

**Figure 4.**
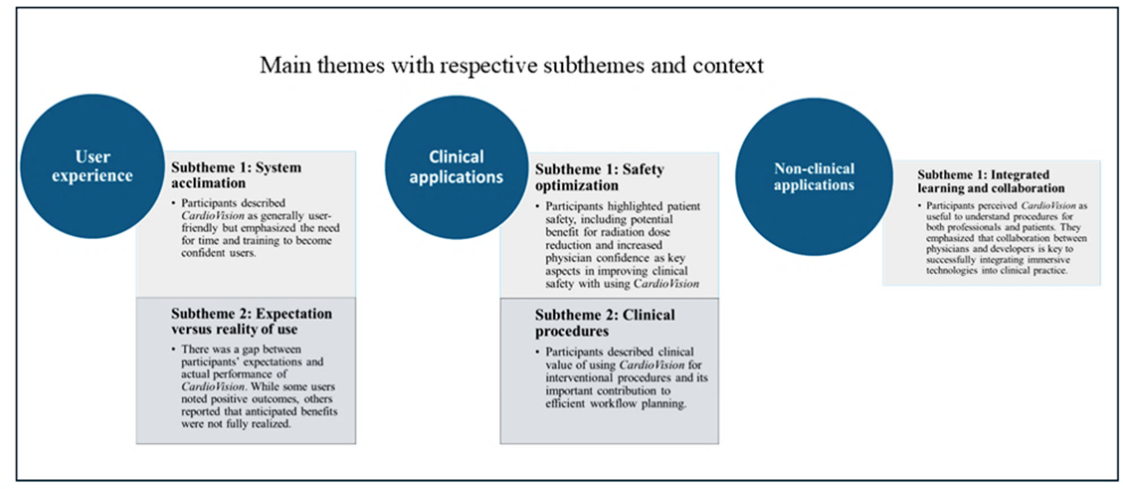
Emerging themes from interview data.

In the data from the interviews, three main themes, user experience, applications, clinical interventions, and nonclinical interventions, emerged, with subthemes and related categories (Figure 4).

### User Experience

The theme user experience comprises two subthemes: the ease with which participants became familiar with the software (system acclimation) and the alignment between their expectations and their actual experience. In general, most of the specialist physicians found the software easy to use. The specialist physicians expressed that the software was intuitive and suitable for clinical use. Most participants during the interviews expressed the value of the *CardioVision* software’s use in clinical practice as from the statements below:

*No, it was, as I said, a very nice experience...the benefits of it and what I saw, I mean, you don't need a lot of cases to be able to appreciate, maybe not judge, but to appreciate what such a tool can bring you in everyday practice.*

[PT 6]

*I mean, the whole experience, especially the way you change your way of thinking, you know, as you put it inside the procedure you are going to do. That’s something I really liked.*

[PT 5]

Although overall usability was considered acceptable, the results point to areas for improvement and emphasize the importance of allowing users time to adapt to the software in clinical practice, as reflected in the participants’ comments below:

*...I mean, if you're talking about things to continue working on it, so then the cross-sections, and I guess the other thing to try to find the way to incorporate very procedural imaging, like real-time in the software, those are, I think, the two...things to work on i guess in the future*

[PT 2]

*Most of these systems, and that’s no matter which software is behind it, (..) take some time and that will be the most issue I think for clinicians in using it on daily practice*

[PT1]

The specialist physicians express both unfulfilled and positive outcomes. Some specialist physicians noted limitations, such as difficulty recognizing nearby body implant devices within the area of pathology, segmentation issues, and the need for higher resolution. They also highlighted the importance of precise tissue differentiation. On the positive side, the system successfully simulated procedures like stent implantation and provided excellent visualization for large vascular structures. Some specialist physicians found the *CardioVision* software to be more immersive, providing more details, and was perceived as beneficial when compared to other software they currently use in clinical practice.

### Applications: Clinical Interventions

Applications in clinical interventions included two subthemes: safety optimization and value for clinical procedures. The specialist physicians suggested that the software could be useful to optimize general patient safety, optimize radiation dose, and reinforce physician confidence. Enhanced anatomical visualization through *CardioVision* software was suggested to improve safety optimization by enabling physicians to perform IC procedures with greater precision and efficiency. In value for radiation dose optimization, most of the specialist physicians highlighted the reduction in time the software could potentially provide when performing procedures. Some, however, highlighted a trade-off between using CT scans to acquire imaging data for use in the *CardioVision* software and the low radiation dose currently used in procedures of pediatrics, noting that while CT provides quality imaging, it requires radiation, which may offset its benefits, as stated below:

*...with standard catheterization labs nowadays. they are really very low dose already. If we look at the doses that we use in small kids, the doses have been reduced already quite a lot. So I think if you probably add a CT scan, that’s probably much higher than what we use in a standard procedure. Of course, sometimes there are very long procedures where we are balancing the right position. And maybe in some cases, you might be able to reduce radiation....But I'm not...completely convinced that in general you will be able to achieve a dose reduction in all the cases that you want to use*

[PT2]

Additionally, better imaging and anatomical visualization were suggested to boost clinicians’ confidence, contributing to better planning and smoother IC procedures as expressed in a statement of one participant.

*I think CardioVision added something because...you can see better all the images, so I feel more comfortable of the procedure, and I think in this particular case I planned better my procedure. More confidence and also maybe a little...more faster...during the procedure...i didn't measure but It was my feeling*

[PT4].

In the subcategory of clinical procedures, the participants pointed out specific areas where they perceived the software to be of value in clinical practice. This included value across all general types of IC procedures and for workflow planning. Workflow planning included facilitating the selection of appropriate medical devices before scheduling the patient for the procedure.

*The time is not only the time you are in the cath lab, but also the time you prepare the case. But it’s maybe bigger than the time in the cath lab. And also, if you prepare well, you will have a shorter time in cath lab. So, I think this is the general practice of every interventional cardiologist. So you go in the cath lab, you don't want to have a surprise. We don't like surprise in general. So we want to have all the material ready, for example, because not all the calf lab has every kind of stent, balloon, valve, etc*.

[PT4]

The participants further specifically highlighted the software’s potential in preplanning and performing of surgical procedures as highlighted by a pediatric cardiologist below.

*...in most cases where I use 3D segmented images is actually planning of complex surgical repairs...or complex congenital heart disease where the surgeon needs to... [suture] in a certain patch. And so the experience that I have is always focused on that. I think the platform could also be used for that...I mean, if you have a proper...VR platform where you can cut through and turn around images you could also use it for surgical planning of complex heart disease*

[PT2]

### Applications: Nonclinical Interventions

The theme applications for nonclinical interventions focused on one subtheme: integrated learning and collaboration. This included collaboration among health care professionals, patients, and software developers. All participants agreed that the software could be a valuable tool for educating medical students. Some participants also highlighted its usefulness in collaborative discussions with both patients and other healthcare professionals. Others emphasized its potential for discussing treatment plans and diagnostic outcomes with patients. Additionally, some participants stressed the importance of engagement between specialist physicians and software developers, noting that this collaboration could help bridge the gap between clinical needs and available technology. Testing the software’s performance together with the developer for easier integration into clinical workflow was also highlighted as vital when considering adaptation of such immersive technologies in IC practices.

…, *I think it was something very interesting from my experience. And I think also for [developer], because...what we think is important, sometimes an engineer or a person that do another job don't know that this can be important in operating theater. So, I think the cooperation was very interesting from both side*

[PT3]

### Specialist Physicians’ Overall Satisfaction With Use of Software

Table 4 below shows the reported areas of satisfaction by the specialist physicians. The highest positive feedback was in the visualization of structures, where all users agreed or strongly agreed (100%). Decision-making and preparation of complex cases also received positive feedback, with ratings of agree/strongly agree at 75% and 63%, respectively. A slight variation was, however, noted in use for preparing complex cases, as others were neutral (25%) or disagreed with this (13%). Half of the participants (50%) reported being satisfied with the user functions, while the other half (50%) gave neutral responses, indicating potential areas for improvement.

**Table 4.** Specialist physicians (n=8) rated areas of satisfaction with *CardioVision.*

Rated areas of satisfaction	Strongly disagree, n (%)	Disagree, n (%)	Neutral, n (%)	Agree, n (%)	Strongly agree, n (%)
User functions	0 (0)	0 (0)	4 (50)	2 (25)	2 (25)
Prepare complex cases	0 (0)	1 (12.5)	2 (25)	4 (50)	1 (12.5)
Visualization of structures	0 (0)	0 (0)	0 (0)	7 (87.5)	1 (12.5)
Decision making	0 (0)	0 (0)	2 (25)	4 (50)	2 (25)

## Discussion

### Principal Findings

This study aimed to assess the experiences of specialist physicians in IC in using a pediatric IC immersive technology, *CardioVision*. The focus was on how the specialists perceived the benefits of the technology in relation to IC procedures and other relevant applications. The discussion adopts a clinical perspective, evaluating the balance between expectations and actual experiences with immersive technology such as *CardioVision*. It also identifies areas for improvement, particularly those that address the unique clinical needs of IC specialist physicians.

A key motivation for most specialist physicians in using *CardioVision* in our study was the need to enhance clinical value during IC procedures. Cardiac anatomy is complex, and visualizing structural deformities presents a significant challenge [3,27]. Immersive technologies have shown great promise in improving procedural accuracy, efficiency, and clinical decision-making for cardiac pathologies in both adult and pediatric populations [5,6,28]. A central finding from our study is that IC specialist physicians highly valued the ability of immersive technology to deliver precise and accurate visualization of cardiac anatomy. In a study to assess assistance with diagnosis, Lee et al [29] demonstrated that immersive technology was the most effective tool for visualizing CHD and anatomical comprehension. Studies, however, report that visual quality is one of the most demanding aspects of immersive technology with image display, often representing the largest design and cost constraint [5,30]. In IC, the precision of visual information is paramount [7]. High-resolution displays are crucial for accurately visualizing complex cardiac structures, aiding in diagnosis and procedural planning [31,32]. Our study indicates that immersive technologies that add clinical value, support procedural precision, and improve the quality of pathological visualization are highly valued by IC specialist physicians. This perceived usefulness is a critical factor in their willingness to adopt such technologies in clinical practice [29], underscoring its role in facilitating successful implementation and guiding future development in IC.

The technology’s time efficiency also emerged as a crucial consideration for the IC specialist physicians in our study. Increased time efficiency had two primary implications: decreasing radiation exposure during procedures and improving workflow efficiency in already demanding clinical settings. The specialist physicians in our study noted only an indirect link between the use of *CardioVision* and reduced radiation exposure, suggesting that increased precision and shorter procedure times could potentially contribute to dose reduction. Supporting this is a review by Samant et al [8] that highlights how immersive technologies, specifically VR and AR, enhance visualization of complex structural heart defects, leading to reduced procedural times and radiation exposure. Park et al [33] also report that an AR software used in CT-guided needle procedures improved accuracy, reduced procedure time, and lowered radiation exposure, including enabling less experienced health care professionals to perform comparably to experts. The specialist physicians in our study further emphasized the need for ease of use and a minimal learning curve to ensure quick adoption of immersive technologies. Many immersive technologies will require some time invested to learn and integrate into clinical workflows [4]. The time needed to learn the software is important to consider when deciding to integrate it into clinical practice to minimize disruptions in busy IC departments.

*CardioVision* was perceived by the specialist physicians in our study as a valuable decision-support tool, particularly in complex IC procedures. The reported current applications for immersive technologies include diagnosis, preoperative planning, communication, and patient management [34]. Decision support, as highlighted in our study, also extended to workflow planning aspects such as preprocedure device selection. This is of particular importance in pediatric IC, where appropriately sized stents, catheters, and other essential devices may be scarce. Device limitations are reported to persist due to the lack of designs tailored to infants [35]. The specialist physicians in our study highlighted that improved planning and visualization tools, such as those offered by *CardioVision*, fostered greater procedural preparedness, minimized unexpected challenges, and enhanced physician confidence. Studies also report that immersive technologies contribute positively to physician confidence during medical procedures [4,36]. A study by Robertson et al [37] showed that VR modeling for surgical procedures increased surgeon confidence and, in some cases, altered the planned approach, with surgeons reporting improved mental preparation and a sense of familiarity during operations.

The *CardioVision* software was also seen as a facilitator of multidisciplinary collaboration, enabling health care professionals to jointly evaluate patient-specific cases. A study by Kim et al [38] reports preference in the use of VR for collaborative case discussions, emphasizing this role of immersive technologies. The specialist physicians in our study also appreciated the *CardioVision’*s potential to improve communication with patients to better understand their condition and treatment options. This is a benefit of immersive technologies reported in several studies [34,39,40]. For example, a study by El Mathari et al [40] showed that using VR for patient education enhanced satisfaction by delivering information in a way that patients found more informative and engaging. The *CardioVision* was also recognized by all the specialist physicians in our study as highly valuable for training and education. In education, the benefits of immersive technologies include offering realistic simulations of complex cardiac procedures and complementing traditional training methods by enabling students’ repeated practice on procedures [4,41,42].

Integrating immersive technologies into clinical workflows, however, remains a challenge. In our study, the specialist physicians noted that while *CardioVision* holds promise, successful implementation will require careful attention to hardware compatibility, image quality, intuitive user interfaces, and workflow integration. Emphasis was placed on the need for close collaboration with developers to ensure such software meets the specific demands of IC departments. Furthermore, it is important to determine whether the software is designed specifically for clinicians, for patients, or for both [12]. While the *CardioVision* software in our study was primarily developed for physicians, participants also highlighted its potential for adaptation to patient use, particularly to support understanding of their medical condition, although this will require further exploration. Annabestani et al [7] state that collaboration between medical specialists and technical experts is central to advancing the use of immersive technology in cardiac interventions. Co-development between clinicians, engineers, and researchers is essential to maximize clinical relevance and ensure these technologies are both effective and adoptable in routine practice [7]. Through close collaborations, medical imaging processes can be streamlined and imaging timelines accelerated [32], enabling the overall adoption of immersive technologies in routine health care practice. Annabestani et al [7] suggest that close collaboration with technology developers drives innovation by facilitating seamless integration of advanced tools, enabling precise, patient-specific procedures that enhance both safety and clinical outcomes.

### Study Limitations

Our study had several limitations that should be acknowledged. First, the sample size in the quantitative analysis was relatively small, which limited the statistical power to detect meaningful variability or trends in the data. However, the quantitative data were complemented by rich insights obtained from qualitative interviews, which helped to contextualize and strengthen the overall interpretation of results.

Second, the participants had only a limited timeframe to evaluate the *CardioVision* software. This brief exposure may not have been sufficient for users to fully explore the system’s capabilities or to become comfortable with its interface and functions. As a result, their reported experiences and perceived usefulness of the software may not accurately reflect longer-term use scenarios. Additionally, the study was conducted in a controlled environment. The few specialist physicians who participated in the study were from selected countries and health care centers. This may not capture the full complexity and workflow dynamics of routine clinical practice. However, the strategically selected specialist physicians who participated in the study are likely to provide expert opinion of transferable value. Future research with larger sample sizes, extended periods of use, and on-site evaluations across different clinical settings would provide a more comprehensive understanding of the software’s potential and limitations. Our study, however, focuses on highlighting what IC specialist physicians consider valuable in immersive technologies. Using *CardioVision* as a case study helps to identify key areas for consideration when integrating immersive technologies into an IC practice.

### Conclusions

This study highlights that IC specialist physicians perceive immersive technologies like *CardioVision* as valuable tools for enhancing procedural precision, anatomical visualization, and clinical decision-making. The software was described as having the potential to improve workflow efficiency, support preoperative planning, and facilitate multidisciplinary and patient collaboration. Integrating such technologies seamlessly into existing clinical workflows should consider the need for user-friendly interfaces and minimal learning curves to gain confidence for effective use. Future advancements should focus on co-developing solutions with clinicians to ensure that immersive technologies are tailored to meet the specific needs of IC practice, thereby maximizing clinical utility and adoption.

## Supplementary material

10.2196/79278 Multimedia Appendix 1 CardioVision study survey.

10.2196/79278 Multimedia Appendix 2 Interview guide.

## Abbreviations

AI: artificial intelligence
AR: augmented reality
CHD: congenital heart disease
CT: computed tomography
HARMONIC: health effects of cardiac fluoroscopy and modern radiotherapy in pediatrics
IC: interventional cardiology
MR: mixed reality
SUS: System Usability Scale
VR: virtual reality
