# Supplementary material for: Usability Evaluation of a Mixed Reality Platform in Pediatric Interventional Cardiology by Specialist Physicians: Mixed Methods Study
Source: JMIR Form Res. 2025 Nov 19;9:e79278. doi: 10.2196/79278 (PMC12629521; doi:10.2196/79278)
Supplement: Multimedia Appendix 1 [file formative-v9-e79278-s001.pdf]

Nettskjema

Specialist physicians experience with Pre-Planning of Clinical Cases Using the CardioVision Platform

Are you interested in taking part in the research project:

Specialist physicians' Experience with Pre-Planning of clinical cases in interventional radiology using the CardioVision Platform

Purpose of the project
You are invited to participate in a research project where the main purpose is to determine: what features Specialist Physicians emphasis as vital in supporting acceptability and user experience when testing Mixed Reality (MR)/Virtual Reality (VR) software such as the *CardioVision* Platform in preparing for interventional radiological procedures.

Objectives
The objective of the project is to determine the relevance and importance of different features of the *CardioVision* platform in the context of real clinical cases and medical procedure preparation.The research question explores which features specialist physicians emphasis as vital in supporting acceptability and user experience when testing MR/VR software in preparing for medical procedures. This is a research project that builds on the delivery aspect of theHealth Effects of Cardiac Fluoroscopy and Modern Radiotherapy in Pediatrics (HARMONIC) project.The data collected will contribute to a scientific publication and a comprehensive report on the software's performance, further supporting the goals of the HARMONIC project.

Which institution is responsible for the research project?
The University of Southeastern Norway (USN) is responsible for the project (data controller).

Why are you being asked to participate?
We are inviting you to participate in our research project, which focuses on understanding the features of the CardioVision Mixed Reality /Virtual Reality software to assess specialist physician's user experience and acceptability during medical procedure preparation. You have been selected/ targeted for participation as a specialist physician with experience in interventional radiological procedures and the use of MR/VR technologies. You are receiving this invitation because your qualifications align with our selection criteria, and we believe your insights will greatly contribute to our understanding of how CardioVision can be optimized to support clinical workflows and enhance patient care in interventional radiology.

What does participation involve for you?
In this research project the data collection uses a mixed method approach i.e use of an online questionnaire survey and interviews. Only data related to your profession will be collected. In the online survey after giving consent, you will be transferred to a link to start the survey below. Upon completion of the survey, a date and time will be setup for a follow up interview. Both the survey and questionnaire will only constitute questions related to your professional area of experience and use of the CardioVision. In the survey your responses will be collected online. The interview will be recorded digitally.No other information will be collected and processed other than that which you have provided.

Participation is voluntary
Participation in the project is voluntary. If you chose to participate, you can withdraw your consent at any time without giving a reason. All information about you will then be made anonymous. There will be no negative consequences for you if you chose not to participate or later decide to withdraw.

Your personal privacy – how we will store and use your personal data

Nettskjema

We will only use your personal data for the purpose(s) specified here and we will process your personal data in accordance with data protection legislation (the GDPR). I,e The raw data will only be accessed by the unauthorized persons. The recorded data will be transcribed. All personal data will be replaced with a code. The list of names, contact details and respective codes will be stored separately from the rest of the collected data. The recorded data will be deleted after transcribing. All participants will not be identifiable at publication.

What will happen to your personal data at the end of the research project?

The planned end date of the project is [6 September 2025]. The audio collected data will be deleted. Any the transcribed data with personal data anonymised will be stored. The data from the questionnaire will also be stored as anonymised data.

Your rights

So long as you can be identified in the collected data, you have the right to:
Access the personal data that is being processed about you
Request that your personal data is deleted
Request that incorrect personal data about you is corrected/rectified
Receive a copy of your personal data (data portability), and
Send a complaint to the Norwegian Data Protection Authority regarding the processing of your personal data

What gives us the right to process your personal data?

We will process your personal data based on your consent.
Based on an agreement with the University of South eastern Norway (USN). The Data Protection Services of Sikt – Norwegian Agency for Shared Services in Education and Research has assessed that the processing of personal data in this project meets requirements in data protection legislation.

Where can I find out more?

If you have questions about the project, or want to exercise your rights, contact:
University of south-eastern Norway
Project leader; Catherine Chilute Chilanga: email: catherine.chilanga@usn.no
Our Data Protection Officer: Contact email: personvernombudet@usn.no
If you have questions about how data protection has been assessed in this project by Sikt, contact: email: (personverntjenester@sikt.no)
Yours sincerely,
Catherine C Chilanga
Project Leader
(Researcher)

I have received and understood information about the project [Specialist physicians' Experience with Pre-Planning of Clinical Cases Using the CardioVision Platform] and have been given the opportunity to ask questions. I give consent

I consent to participate

I do not consent

Participant Characteristics

1. Specify your area of medical specialty

2. How many years of experience do you have in working with interventional radiological procedures?

1-5 years

6-10 years

Over 10 years

3. Does your experience include pediatric cardiac procedures

Yes

No

4. Where have you primarily practiced clinically to date?

Public practice

Private practice

Other

If other: Please specigy

5. Are you familiar with Mixed Reality/ Virtual Reality (MR/VR) technologies used in clinical settings?

Yes

No

6. If you are familiar with MR/VR technologies, have you used them for cardiology procedures in your practice?

Yes

No

No familar with MR/VR technologies

User expectation of the software

On a scale from 1 to 5, where 1 is strongly disagree and 5 is strongly agree, how would you rate your agreement with the following statements about the expected value of the CardioVision software during interventional radiological procedures?

Rate the following on a scale from 1 to 5, where 1 is strongly disagree and 5 is strongly agree

1. I expect that the software to be user-friendly.

1. Strongly disagree
2. Disagree
3. Neutral
4. Agree
5. Strongly agree

2. I expect the software to integrate well with other tools

1. Strongly disagree
2. Disagree
3. Neutral
4. Agree
5. Strongly agree

3. I expect the software to facilitate planning of complex interventional radiological procedures.

1. Strongly disagree
2. Disagree
3. Neutral
4. Agree
5. Strongly agree

4. I expect the software to enhance efficiency of performing procedures

1. Strongly disagree
2. Disagree
3. Neutral
4. Agree
5. Strongly agree

5. I expect that the software to assist in decision- making for individual procedures.

1. Strongly disagree
2. Disagree
3. Neutral
4. Agree
5. Strongly agree

Please specify any other expectations you have.

Part A: Standard System Usability Scale (SUS)

On a scale from 1 to 5, where 1 is strongly disagree and 5 is strongly agree, please rate your agreement with the following statements about the usability of the CardioVision software.

Rate the following on a scale from 1 to 5, where 1 is strongly disagree and 5 is strongly agree

1. I think that I would like to use this software frequently.

1. Strongly disagree
2. Disagree
3. Neutral
4. Agree
5. Strongly agree

2. I found the software unnecessarily complex.

1. Strongly disagree
2. Disagree
3. Neutral
4. Agree
5. Strongly agree

3. I thought the software was easy to use

1. Strongly disagree
2. Disagree
3. Neutral
4. Agree
5. Strongly agree

4. I think that I would need the support of a technical person to be able to use this software.

1. Strongly disagree
2. Disagree
3. Neutral
4. Agree
5. Strongly agree

5. I found the various functions in this software were well integrated.

1. Strongly disagree
2. Disagree
3. Neutral
4. Agree
5. Strongly agree

6. I thought there was too much inconsistency in this software

1. Strongly disagree
2. Disagree
3. Neutral
4. Agree
5. Strongly agree

7. I would imagine that most people would learn to use this software very quickly.

1. Strongly disagree
2. Disagree
3. Neutral
4. Agree
5. Strongly agree

8. I found the software very cumbersome to use.

1. Strongly disagree
2. Disagree
3. Neutral
4. Agree
5. Strongly agree

9. I felt very confident using the software

1. Strongly disagree
2. Disagree
3. Neutral

4. Agree
5. Strongly agree

10. I needed to learn a lot of things before I could get going with this software

1. Strongly disagree
2. Disagree
3. Neutral
4. Agree
5. Strongly agree

Part B: Additional Usability Questions

1. General Usability

On a scale from 1 to 5, where 1 is strongly disagree and 5 is strongly agree, how would you rate your agreement with the following statements about the usability of the CardioVision software?

Rate the following on a scale of 1 to 5, where 1 is strongly disagree and 5 strongly agree

1. I found most of the software features to be user-friendly.

1. Strongly disagree
2. Disagree
3. Neutral
4. Agree
5. Strongly agree

2. The software provided adequate assistance in preparing difficult interventional radiological cases

1. Strongly disagree
2. Disagree
3. Neutral
4. Agree
5. Strongly agree

3. I was able to accomplish the procedure within the reasonable expected time frame using the software

1. Strongly disagree
2. Disagree
3. Neutral
4. Agree
5. Strongly agree

4. I found the software features useful for improving my workflow

1. Strongly disagree
2. Disagree

3. Neutral
4. Agree
5. Strongly agree

5. I believe additional features could enhance the usability of the software

1. Strongly disagree
2. Disagree
3. Neutral
4. Agree
5. Strongly agree

2. Usefulness for Cardiac Procedures

On a scale from 1 to 5, where 1 is strongly disagree and 5 is strongly agree, how would you rate your agreement with the following statements about the usefulness of the CardioVision software for interventional radiological procedures?

Rate the following on a scale from 1 to 5, where 1 is "Strongly Disagree" and 5 is "Strongly Agree

1. The software provided better visualization of anatomical structures.

1. Strongly disagree
2. Disagree
3. Neutral
4. Agree
5. Strongly agree

2. The software provided precision in targeting pathology

1. Strongly disagree
2. Disagree
3. Neutral
4. Agree
5. Strongly agree

3. The software enhanced my ability to plan complex procedures

1. Strongly disagree
2. Disagree
3. Neutral
4. Agree
5. Strongly agree

4. The software provided valuable support for my decision-making strategies.

1. Strongly disagree
2. Disagree
3. Neutral
4. Agree

5. Strongly agree

5. The software reduced the overall time required to perform the procedures

1. Strongly disagree
2. Disagree
3. Neutral
4. Agree
5. Strongly agree

Satisfaction Level

On a scale from 1 to 5, where 1 is "Strongly Disagree" and 5 is "Strongly Agree," please indicate your level of agreement with the following statements about the CardioVision software.

Rate the following on a scale from 1 to 5, where 1 is "Strongly Disagree" and 5 is "Strongly Agree

1. I am satisfied with the user functions provided by the software

1. Strongly disagree
2. Disagree
3. Neutral
4. Agree
5. Strongly agree

2. I am satisfied with the software's capability in preparing challenging interventional radiological cases

1. Strongly disagree
2. Disagree
3. Neutral
4. Agree
5. Strongly agree

3. I am satisfied with the software’s ability to visualize anatomical structures and pathology

1. Strongly disagree
2. Disagree
3. Neutral
4. Agree
5. Strongly agree

4. I am satisfied with the software's ability to assist with decision-making strategies for specific medical cases

1. Strongly disagree
2. Disagree
3. Neutral
4. Agree
5. Strongly agree

Nettskjema

Feedback on Features

Please describe any features of the CardioVision software that you found particularly important for supporting simulation of interventional radiological procedures.

Outline some questions that you would like to discuss in the interview

Please provide your name and preferred email address. We will use this to contact you to arrange for an interview. Thank you for your participation
