# Supplementary material for: Usability Evaluation of a Mixed Reality Platform in Pediatric Interventional Cardiology by Specialist Physicians: Mixed Methods Study
Source: JMIR Form Res. 2025 Nov 19;9:e79278. doi: 10.2196/79278 (PMC12629521; doi:10.2196/79278)
Supplement: Multimedia Appendix 2 [file formative-v9-e79278-s002.pdf]

Interview Guide: Assessing the CardioVision Platform

Introduction (5 Minutes)

1. **Purpose:** We are here to discuss your experiences with the CardioVision platform and gather feedback that could help improve the software.
2. **Voluntariness:** Your participation is entirely voluntary, and you may withdraw at any time. (Consent obtained in survey)
3. **Data Security:** All your responses will be kept confidential and used solely for research purposes.
4. **Interview Structure:** We will cover several themes, each focusing on different aspects of your experience with the CardioVision platform.
5. **Questions:** Do you have any questions before we begin?

Theme 1: Initial Impressions and Setup (5 Minutes)

1. Can you describe your initial impressions of the CardioVision software when you first used it?
2. Once the system was set up, how intuitive or straightforward was it for you to start using the simulator?
3. Did you encounter any issues or difficulties while using the simulator after the setup? If so, what were they?
4. Are there any suggestions you have for improving the user experience or resolving any issues that you encountered?

Keywords: First impressions, ease of use, setup, technical issues.

Theme 2: Experience Using the CardioVision Platform (10 Minutes)

1. How would you describe your experience using the CardioVision platform for planning of procedures?
2. Can you provide an example of a specific procedure where the software was particularly useful?
3. How realistic did you find the software in comparison to actual procedures you perform?

Keywords: Realism, usability, case-specific examples.

Theme 3: Performance of CardioVision platform (5 Minutes)

1. Did the software meet your expectations in terms of visualizing anatomical structures and pathology in complex cases?
2. How would you describe your experience with the software’s performance in for example:
   - Visualizing areas of pathology?
   - Assisting with the insertion of necessary devices (e.g., catheters)?
   - Conducting and completing the procedure?
   - Other?

3. Can you provide an example of a case where the software successfully helped achieve dose optimization?
4. What improvements do you think could be made to enhance the software’s performance?

Keywords: Structure/pathology visualization, dose optimization, User acceptance.

Theme 4: Future Enhancements and Features (5 Minutes)

1. What features did you find most valuable in the Cardio-Vision platform?
2. Are there any additional features you would like to see added to the software?
3. How do you think these new features could improve the experience for you and other users?
4. Were there any aspects of the current software that you felt were lacking or needed improvement? Please explain.

Keywords: Future features, improvements, achieved expectations.

Theme 5: Overall Satisfaction and Impact (5 Minutes)

1. Overall, how satisfied are you with your experience using the CardioVision platform?
2. How do you think this tool will impact your practice in the long term?
3. Would you recommend this platform to other medical specialist/cardiologists? Why or why not?
4. Did the software meet your expectations? How and why?
5. If you could communicate one key message to the developers of the Cardio-Vision platform, what would it be?

Keywords: Overall satisfaction, long-term impact, recommendations.

Conclusion (5 Minutes)

1. Thank you very much for participating in this interview and sharing your insights.
2. Next steps and how your feedback will be used.
3. How was the experience of participating in this interview?
4. Any final questions or comments?
